# Solar-Driven Photoelectrochemical Performance of Novel ZnO/Ag_2_WO_4_/AgBr Nanorods-Based Photoelectrodes

**DOI:** 10.1186/s11671-021-03586-z

**Published:** 2021-08-21

**Authors:** Elfatih Mustafa, Rania E. Adam, Polla Rouf, Magnus Willander, Omer Nur

**Affiliations:** 1 Department of Sciences and Technology, Linköping University, Campus Norrköping, 601 74 Norrköping, Sweden; 2 Department of Physics, Chemistry and Biology (IFM), Linköping University, 58183 Linköping, Sweden

**Keywords:** ZnO nanorods, Silver tungsten, Silver bromide, Heterojunction, Photoelectrodes, Water oxidation

## Abstract

**Abstract:**

Highly efficient photoelectrochemical (PEC) water oxidation under solar visible light is crucial for water splitting to produce hydrogen as a source of sustainable energy. Particularly, silver-based nanomaterials are important for PEC performance due to their surface plasmon resonance which can enhance the photoelectrochemical efficiency. However, the PEC of ZnO/Ag_2_WO_4_/AgBr with enhanced visible-light water oxidation has not been studied so far. Herein, we present a novel photoelectrodes based on ZnO/Ag_2_WO_4_/AgBr nanorods (NRs) for PEC application, which is prepared by the low-temperature chemical growth method and then by successive ionic layer adsorption and reaction (SILAR) method. The synthesized photoelectrodes were investigated by several characterization techniques, emphasizing a successful synthesis of the ZnO/Ag_2_WO_4_/AgBr heterostructure NRs with excellent photocatalysis performance compared to pure ZnO NRs photoelectrode. The significantly enhanced PEC was due to improved photogeneration and transportation of electrons in the heterojunction due to the synergistic effect of the heterostructure. This study is significant for basic understanding of the photocatalytic mechanism of the heterojunction which can prompt further development of novel efficient photoelectrochemical-catalytic materials.

**Graphic Abstract:**

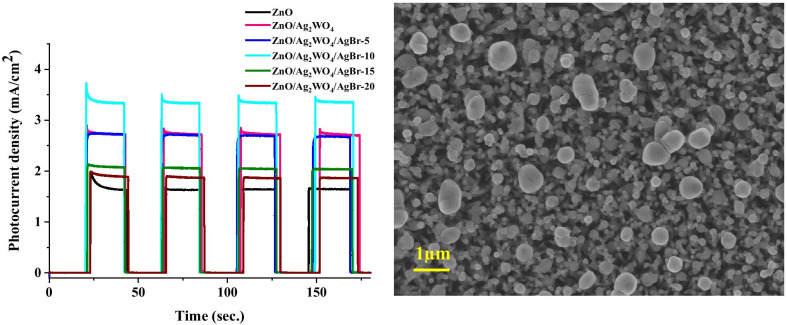

**Supplementary Information:**

The online version contains supplementary material available at 10.1186/s11671-021-03586-z.

## Introduction

Water splitting through photoelectrochemical (PEC) [1–3] processes can provide a solution for energy sustainability by harnessing the driving energy from the sun, which has conclusive beneficial effect on the environment [4–9]. Nanoheterostructure materials have been used in wide-range applications such as gas sensor, solar cell and water splitting for hydrogen production [10–12]. Many metal oxides semiconductors are investigated extensively for PEC applications such as WO_3_, TiO_2_, Fe_2_O_3_, BiVO_4_, Cu_2_O and ZnO [9, 13–18]. Among these metal oxides, ZnO gains a great interest because of its unique properties for PEC applications. ZnO with its wide band gap has a high reduction and oxidation (redox) potentials to drive the photocatalysis reaction, chemically and physically stable; also it is a non-toxic and is abundant [19–22]. Also, ZnO gains high interest because it nucleates in a variety of different nanostructured forms and different methods of growth can be used. However, ZnO has some draw backs which might reduce its utilization as an efficient electrode for PEC using the sun radiation. The ZnO absorption is mainly limited to the UV wavelength, and its high recombination rate is the main factor that reduces its efficiency during a PEC reaction [23]. This problem can be optimized by surface modification of the ZnO by adding another semiconductor such as Ag/Ag_2_WO_4_ to form a new nanocomposite as in our previous work on PEC using ZnO/Ag/Ag_2_WO_4_ photoelectrode [24]. The result showed high PEC performance in comparison with the ZnO which is attributed to the suppression of the high recombination rate and the shift of the absorption toward the visible-wavelength region due to surface plasmonic resonance (SPR) [24]. In spite of the high performance of the ZnO/Ag/Ag_2_WO_4_ due to the deposition of the Ag_2_WO_4_ into the ZnO nanorods (NRs), still the band gap did not utilize enough visible light to yield an efficient PEC electrode. Thus, smaller-band-gap semiconductor, e.g., AgBr, can be deposited onto the ZnO/Ag_2_WO_4_ heterostructure to further increase the absorption of the visible-light wavelength of the solar spectrum, which further improves the PEC efficiency. AgBr can be a good sensitizer because the same Ag-based material of the Ag_2_WO_4_ could give better light absorption and photoelectron transportation with ZnO/Ag_2_WO_4_/AgBr heterostructure.

In this work, hydrothermal growth route followed by successive ionic layer adsorption and reaction (SILAR) method was used to prepare ZnO/Ag_2_WO_4_/AgBr NRs photoelectrode for PEC water splitting analysis. This implies electrode preparation, characterization, PEC experiments and presenting the proposed electron path during the photocatalytic reaction. Ag_2_WO_4_ was deposited into the sample in order to develop plasmonic sensitizer to improve the utilization of the solar power and accelerating the charge carrier transfer. The Ag in the sample with SPR effect can enhances the photocatalytic performance due to the enhancement of the absorption of the solar light. The loading of the AgBr could further enhances the absorption of light in the visible range due to the lower band gap of the AgBr. As far as we know, it is the first time to prepare ZnO/Ag_2_WO_4_/AgBr NRs photoelectrode by deposition of Ag_2_WO_4_ and AgBr on the ZnO NRs using SILAR method for PEC performance. The ZnO/Ag_2_WO_4_/AgBr NRs photoelectrodes were prepared with 5, 10, 15 and 20 SILAR cycles for AgBr amount optimization.

## Experimental Part

### ZnO/Ag_2_WO_4_/AgBr Photoelectrodes Preparation

**Fig. 1:**
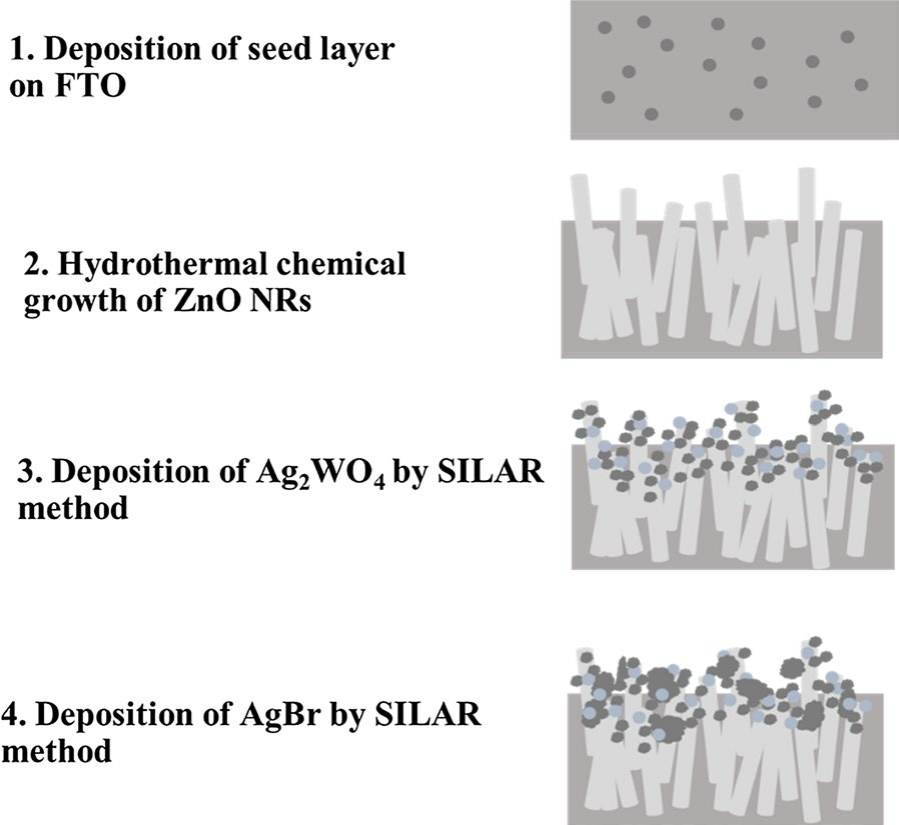
Schematic diagram showing the preparation method of the ZnO/Ag_2_WO_4_/AgBr photoelectrode

The photoelectrodes were prepared using three steps: growth of the ZnO NRs, deposition of Ag_2_WO_4_ and AgBr as illustrated in Fig. 1.

The pristine ZnO NRs were prepared on the FTO covered by a seed layer of ZnO nanoparticles (NPs) prepared by the hydrothermal growth similar to our previous work [24]. The precursor solutions were prepared using equal molecular (0.05 M) of zinc nitrate hexahydrate (Zn (NO_3_)_2_·0.6H_2_O) and hexamethylenetetramine (HMT). Then, the FTO substrates with the seed layer were fixed upside down in a Teflon sample holder and then placed in a beaker containing the growth solution and were kept in a preheated oven at 90 °C for 5 h. After the completion of the growth duration, the samples were taken out and left to cool down to room temperature. Finally, the samples were washed with DI water to avoid any unwanted particles or residuals and then dried with flowing nitrogen gun.

The ZnO/Ag_2_WO_4_ NRs photoelectrode was prepared using SILAR method in the same way as our previous work [24, 25]. An anionic and cationic aqueous precursor solutions were prepared separately using 0.1 M of silver nitrate Ag (NO)_3_ and 0.1 M of sodium tungstate (Na_2_WO_4_.2H_2_O). The deposition took place by immersion of the prepared ZnO NRs samples into the Ag (NO)_3_ solution for 2 min to absorb the silver ions (Ag^+^), and then, they were washed with DI water to remove excess ions or any other unwanted particles. Then, the samples were immersed into the Na_2_WO_4_.2H_2_O solution for 2 min and again washed with DI water. This cycle was repeated several times to obtain enough Ag_2_WO_4_ particles on the ZnO NRs. Then, the samples were dried in an oven at 60 °C for 3 h to obtain good adhesion of the Ag_2_WO_4_ on the ZnO NRs.

The ZnO/Ag_2_WO_4_/AgBr NRs photoelectrode was prepared also using the SILAR method. Anionic and cationic aqueous precursor solutions were prepared separately using 0.1 M of silver nitrate Ag (NO)_3_ and 0.1 M of sodium bromide (NaBr). The deposition took place by immersion of the prepared ZnO NRs sample into Ag (NO)_3_ solution for 2 min to absorb the silver ions (Ag^+^), and then, they were washed with DI water to remove excess ions or any other particles. Then, the sample is immersed into the NaBr solution for 2 min and again washed with DI water. This cycle was repeated for several times to obtain enough AgBr particles on the ZnO/Ag_2_WO_4_ photoelectrode followed by drying in the oven at 60 °C for 3 h for better adhesion of the nanoparticles. Also, the ZnO/Ag_2_WO_4_/AgBr NRs photoelectrodes were prepared with 5, 10, 15 and 20 SILAR cycles for PEC analysis to optimize the AgBr content.

### Characterization

The structural properties of our photoelectrodes were studied using powder X-ray diffraction (XRD) with a Philips powder diffractometer (1729 PW) connected to a Cu K(α) radiation source at the generator voltage of 40 kV and the current of 40 mA. A field-emission scanning electron microscope (FE-SEM) equipped with a Sigma 500 Gemini field emission gun operating at 10 kV was used to investigate the sample morphology. The chemical composition of the samples was investigated by X-ray photoelectron spectroscopy (XPS) using a Kratos AXIS Ultra DLD equipped with a monochromatic Al K(α) X-ray source. CasaXPS software was used to analyze the data. In order to analyze the optical properties, the UV–visible spectroscopy (Perkin-Elmer Lambda 900 system) was used.

### Photoelectrochemical Measurements

The photoelectrochemical performance was studied by three electrode photoelectrochemical measurements using SP-200 potentiostat (Bio-Logic, Claix, France). A platinum (Pt) mesh (as the counter electrode) and a standard silver/silver chloride (Ag/AgCl) in 3 M KCl (as a reference electrode) were used with (0.1 M) of sodium sulfate (Na_2_SO_4_) electrolyte. The total immersed area of the electrode in the electrolyte was 1 cm $\times$ 1 cm. The visible-light radiation was obtained by a solar simulator that uses a 100-W ozone free xenon lamp with an output power of 1 sun (AM 1.5).

## Result and Discussion

### Characterization Analysis

**Fig. 2:**
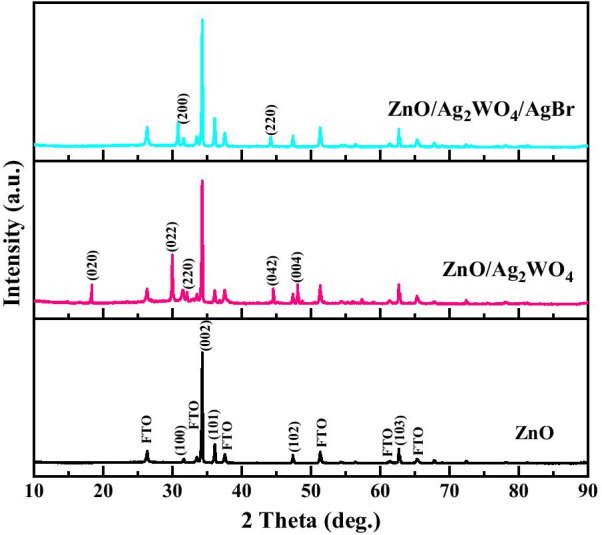
XRD patterns of ZnO, ZnO/Ag_2_WO_4_ and ZnO/Ag_2_WO_4_/AgBr photoelectrodes

Figure 2 shows the XRD spectra of ZnO, ZnO/Ag_2_WO_4_ and ZnO/Ag_2_WO_4_/AgBr samples. It is clear that all the obtained XRD diffraction peaks in the ZnO sample correspond to the hexagonal wurtzite of ZnO (JCPDS no. 36-1451) suggesting no other phases of ZnO were observed. In the XRD pattern of the ZnO/Ag_2_WO_4_ sample, additional peaks were identified, which belonged to Ag_2_WO_4_ (JCPDS no 33-1195), indicating the successful deposition of the Ag_2_WO_4_ into the ZnO NRs. In the XRD pattern of the ZnO/Ag_2_WO_4_/AgBr heterostructure, the planes (200) and (220) labeled in the figure were assigned to the AgBr (JCPDS no 06-438), confirming the formation of AgBr on the ZnO/Ag_2_WO_4_ heterostructure (Additional file 1).

**Fig. 3:**
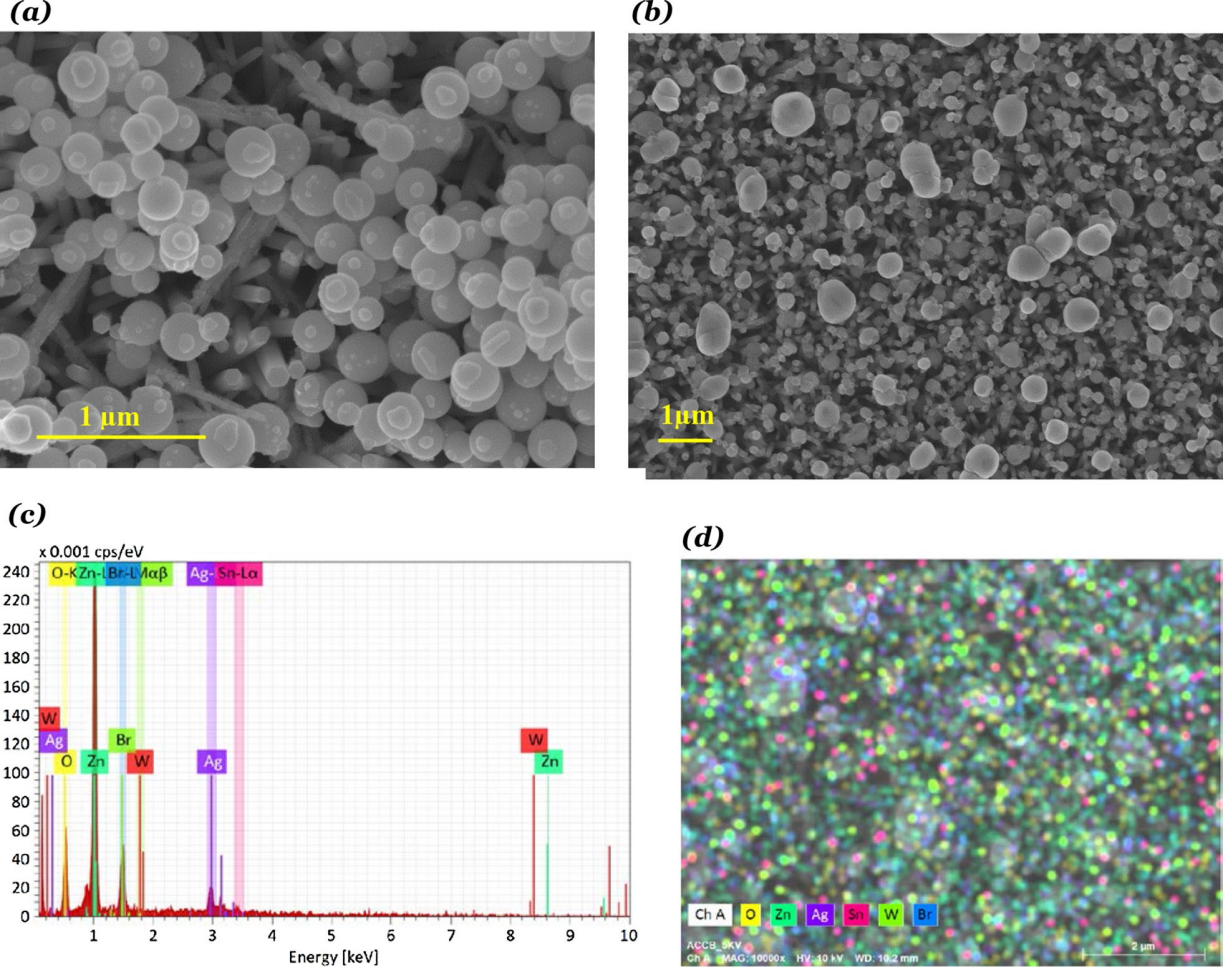

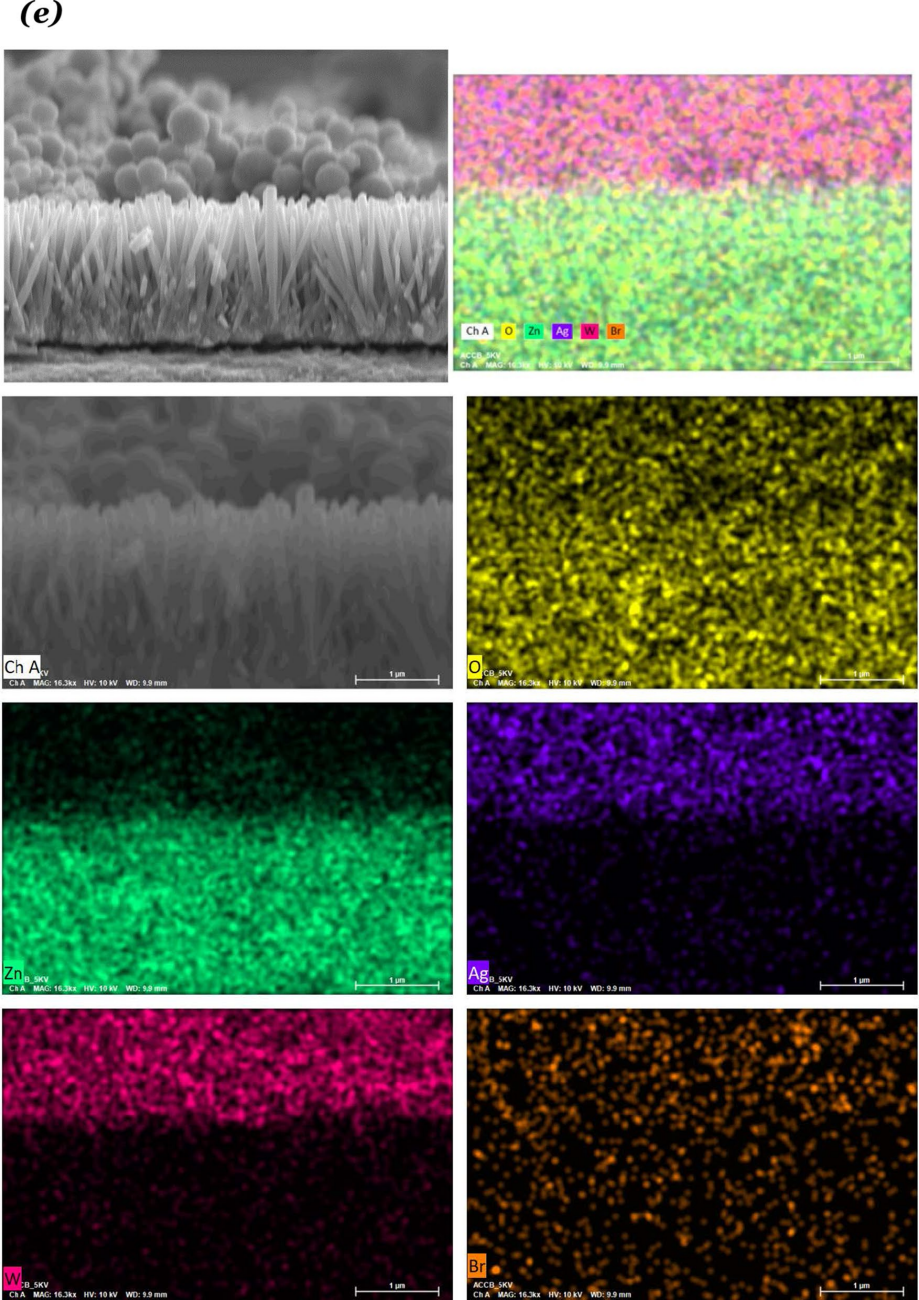
**a** SEM Image of ZnO/Ag_2_WO_4_, **b** SEM Image of ZnO/Ag_2_WO_4_/AgBr, **c** EDX spectrum of ZnO/Ag_2_WO_4_/AgBr photoelectrode, **d** EDX mapping of the ZnO/Ag_2_WO_4_/AgBr heterostructure, and **e** Cross-sectional SEM images and mapping of the ZnO/Ag2WO4/AgBr (10 SILAR cycles) photoelectrode

**Fig. 4:**
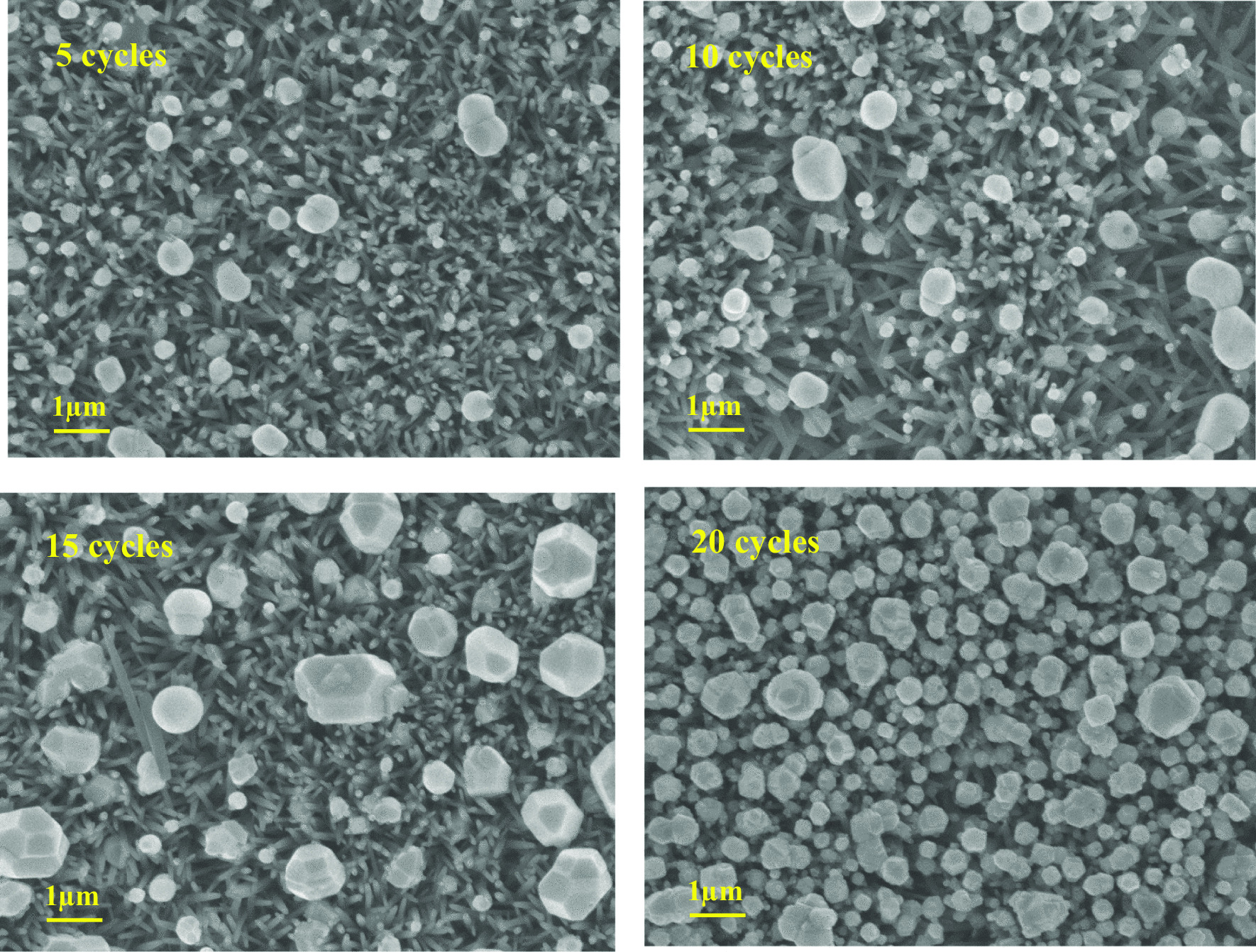
**a** SEM images of the ZnO/Ag_2_WO_4_/AgBr with different amounts of AgBr loading

The FE-SEM images of the ZnO/Ag_2_WO_4_ and ZnO/Ag_2_WO_4_/AgBr NRs photoelectrodes are presented in Fig. 3a, b, respectively. In Fig. 3a, we could clearly see that Ag_2_WO_4_ particles were deposited onto the ZnO NRs. In Fig. 3b, we can notice that a larger particle size is shown for the ZnO/Ag_2_WO_4_/AgBr sample compared to the ZnO/Ag_2_WO_4_ NRs photoelectrode, with semi-crystal structure suggesting the successful deposition of the AgBr onto the sample. Also, tiny particles can be observed, which could be due to different counterpart deposited onto the ZnO NRs. Figure 3c shows EDX spectrum of the ZnO/Ag_2_WO_4_/AgBr NRs photoelectrode which display the relative amount of Zn, O, Ag, W and Br. Surprisingly, unexpected Cl element was detected which is attributed to contamination. In addition to that, the EDX mapping is displayed in Fig. 3d to show the elemental distribution over the sample. Figure 3e shows cross-sectional SEM image and mapping of the ZnO/Ag2WO4/AgBr photoelectrode. It is worth to note that good elemental distribution on the sample could result in high-quality heterostructure yielding an enhanced PEC performance. Moreover, the effect of the AgBr loading on the morphology was investigated for the ZnO/Ag_2_WO_4_/AgBr samples with different cycles of AgBr loading as shown in Fig. 4. As the loading cycles increases, more AgBr particles are deposited onto the sample, reaching full coverage of the surface by the AgBr with 20 cycles of loading cycles.

**Fig. 5:**
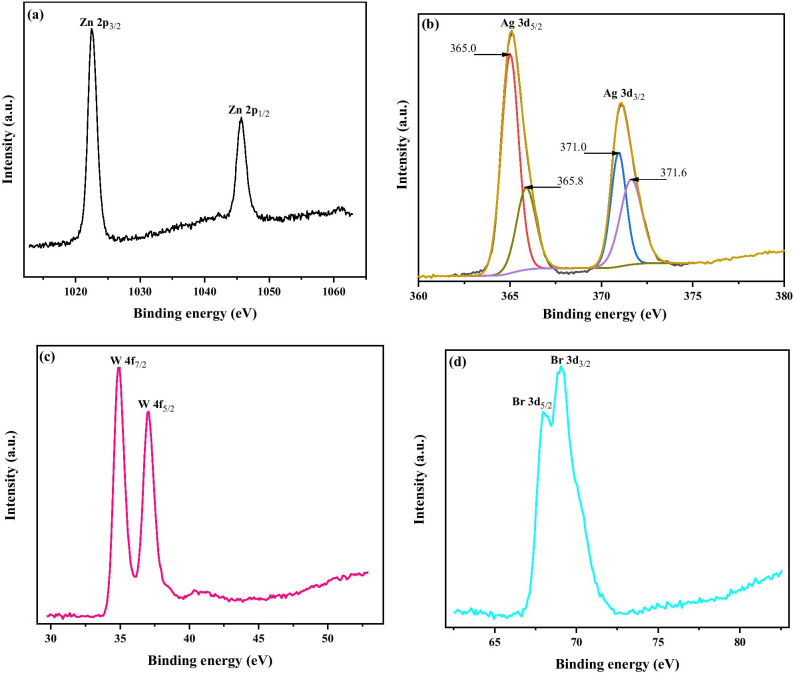
The high-resolution XPS spectrum for Zn, Ag, W and Br collected for the ZnO/Ag_2_WO_4_/AgBr sample

To further evaluate the ZnO/Ag_2_WO_4_/AgBr NRs heterostructure, XPS measurement was taken on all elements present in the structure. Different high-resolution XPS (HR-XPS) spectra are shown in Fig. 5. The Zn 2p spectrum shows two peaks which matches to Zn 2p_3/2_ and Zn 2p_1/2_ with a peak position at 1022.5 and 1045.7 eV, respectively [26, 27], as shown in Fig. 5a. The Ag 3d spectrum is also divided into two peaks with a peak position at 365.1 and 371.1 eV which is attributed to Ag 3d_5/2_ and Ag 3d_3/2_, respectively, as shown in Fig. 5b. The Ag 3d_5/2_ is then divided into two different peaks at 365.0 and 365.8 eV, and the Ag 3d_3/2_ peak is also divided into two different peaks at 371.0 and 371.6 eV.

The peaks at low energies 365.0 and 371.0 eV are assigned to the Ag^+^ in AgBr, whereas the peaks at higher energies 365.8 and 371.6 are assigned to metallic Ag^0^ [28, 29]. Figure 5c shows the spectrum for W 4f which has two peaks positioned at 35.0 and 37.1 eV corresponding to W 4f_7/2_ and W 4f_5/2_, respectively [29]. The Br 3d also exhibits two separate peaks positioned at 68.0 and 69.0 eV assigned to Br 3d_5/2_ and Br 3d_3/2_, respectively, as shown in Fig. 5d [30]. The high-resolution XPS analysis agrees with the EDX measurement and confirms that the intended ZnO/Ag_2_WO_4_/AgBr NRs composite heterostructure has been successfully achieved.

**Fig. 6:**
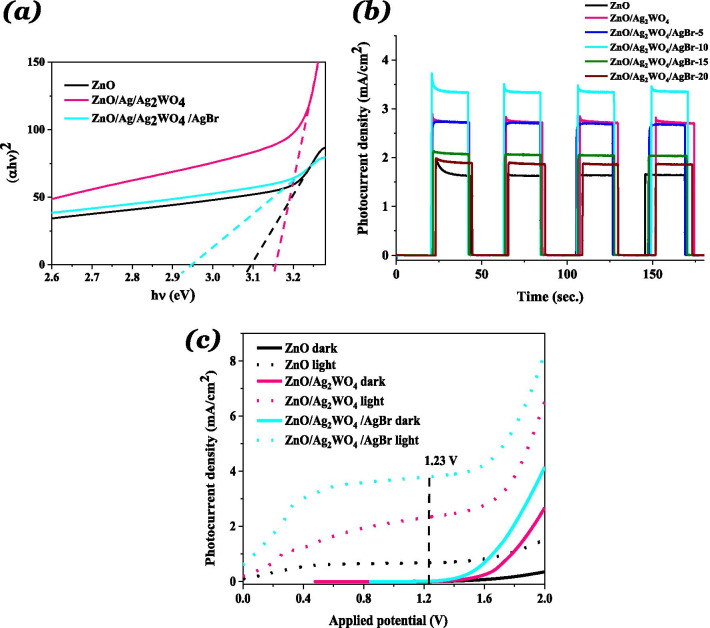
**a** The plots of (αhν)^2^ versus hν, **b** Chronoamperometry I–t curves with solar irradiation on/off cycles,  and **c** LSV curves under dark and visible-light conditions of the ZnO NRs, ZnO/Ag_2_WO_4_ and ZnO/Ag_2_WO_4_/AgBr

From the UV–Vis absorption spectra, a redshift in the optical absorption in the visible-light region was observed for the ZnO/Ag_2_WO_4_/AgBr NRs sample compared to the other samples. Also, the optical band gap energy was reduced to 2.94 eV, which was found from the plots of (αh*v*)^2^ versus photon energy (h*v*) from the UV–Vis absorption spectra as shown in Fig. 6a. The enhancement in the visible-light absorption ability of the ZnO/Ag_2_WO_4_/AgBr NRs heterostructure can be assigned to the deposition of the AgBr due to its lower band gap energy compared to the ZnO. Higher ability to absorb visible light in the heterostructure will be valuable for increasing the efficiency of the PEC reaction.

### Photoelectrochemical Analysis

The PEC properties of the different photoelectrodes investigated by the photoresponse over time of the NRs-based photoelectrodes using chronoamperometry measurements which recorded the photocurrent density versus time in the dark under on/off solar irradiation with an applied potential of + 0.5 V are shown in Fig. 6b. The photocurrent densities were found to be 1.6 mA/cm^2^, for the ZnO photoelectrode which increases up to 2.7 mA/cm^2^ for ZnO/Ag_2_WO_4_ photoelectrode. Further enhancement of the photocurrent density was obtained after the deposition of the AgBr. The enhancement of the photocurrent density is observed when increasing the amount of the AgBr loading with different SILAR cycles. At the lower cycle of 5 times, small amount of photocurrent density (2.7 mA/cm^2^) was detected due to small number of particles that distributed into the surface as it can be seen in the SEM of the 5 time. Increasing the number of cycles to 10 resulted in the highest photocurrent density of 3.3 mA/cm2 with more particles distribution. Further increase of AgBr loading result in a reduction of the photocurrent density to 2 and 1.8 mA/cm^2^ for ZnO/Ag_2_WO_4_/AgBr (15 SILAR cycles) and ZnO/Ag_2_WO_4_/AgBr NRs (20 SILAR cycles) photoelectrodes, respectively, but the interface structure became more uniform size distribution with more crystallization. The possible reason for this reduction is that increasing the AgBr amount leads to form larger aggregates around the ZnO NRs, which might destroy the junctions and reduces the separation of the charge carriers at the interfaces of the heterojunction. Therefore, it is important to optimize the photoelectrodes for higher PEC activity. Due to the best PEC result observed from the ZnO/Ag_2_WO_4_/AgBr NRs (10 SILAR cycles) photoelectrode, this sample is used for all the measurement as optimum photoelectrode.

Linear sweep voltammetry (LSV) measurement was taken under the illumination of solar light and dark conditions at a potential of 1.23 V (vs. Ag/AgCl) electrode. Under the dark condition, a negligible photocurrent density of the ZnO NRs photoelectrode was noticed, which indicates good surface quality of the ZnO NRs as shown in Fig. 6c. In comparison with the ZnO NRs photoelectrode, the ZnO/Ag_2_WO_4_, and the ZnO/Ag_2_WO_4_/AgBr NRs photoelectrodes showed a small photocurrent density under dark condition 0.009 and 0.015 mA/cm^2^, respectively, measured at a potential of 1.23 V indicating an enhancement in the electrical conductivity. Under the illumination of solar light, a lower photocurrent density of 0.7 mA/cm^2^ was observed for ZnO NRs, whereas the photocurrent density was highly increased to 2.3 and 3.8 mA/cm^2^ for the ZnO/Ag_2_WO_4_ and ZnO/Ag_2_WO_4_/AgBr NRs photoelectrodes, respectively. The remarkably improved photocurrent density of the ZnO/Ag_2_WO_4_/AgBr NRs photoelectrode could be ascribed to the heterojunction effect upon the deposition of Ag_2_WO_4_ and the AgBr NPs onto the surface of the ZnO NRs. Which indicates that a high density of the photogenerated electrons can be transferred from the ZnO/Ag_2_WO_4_/AgBr NRs photoelectrode to the counter electrode through the external circuit providing higher photocurrent density. This is attributed to the enhanced charge carrier separation, transportation efficiency, the high absorption of solar light and the shift of the absorption into the visible-light range.

**Fig. 7:**
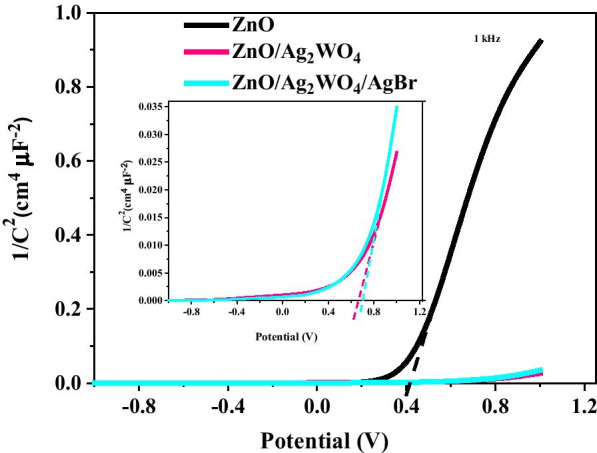
M–S plots of pristine ZnO NRs, ZnO/Ag_2_WO_4_, and ZnO/Ag_2_WO_4_/AgBr photoelectrodes

Mott–Schottky (M–S) analysis is commonly used in PEC for photoelectrodes characterization to understand the electronic properties and the change in the carrier density, which also gives valuable information about the flat band potentials (E_FB_) of the samples [24, 25]. Mott–Schottky plot was obtained by electrochemical impedance measurement at room temperature with selected frequency ⁓ 1 kHz which is based on the capacitance versus applied potential measurements and is shown in Fig. 7. Figure 7 shows the corresponding M–S plots of pristine ZnO NRs, ZnO/Ag_2_WO_4_, and ZnO/Ag_2_WO_4_/AgBr photoelectrodes. It can be seen that all of the prepared samples exhibited positive slopes, showing their n-type nature as expected.

The position of the E_FB_ is estimated by linear extrapolation of the linear region of the curve; the x-axis intercept gives the values of the E_FB_, which were approximately equal to 0.41, 0.70 and 0.74 V for pristine ZnO NRs, ZnO/Ag_2_WO_4_ and ZnO/Ag_2_WO_4_/AgBr NRs photoelectrodes, respectively. Thus, the shift in E_FB_ to higher values could be attributed to the change in charge carrier concentration in the heterojunctions and the change in Helmholtz layer potential [25]. The presence of more surface states can lead to a significant change in the band position that might shift the Fermi level to higher value [24, 31].

Using the dielectric permittivity of the ZnO and the permittivity of the vacuum, the charge carrier density (${N}_{d}$) could be estimated using the following formula [32]:

1 $$N_{d} = \frac{2}{{q\varepsilon \varepsilon_{0} }}\left[ {\frac{1}{{d\left( {1/C^{2} } \right)/dv}}} \right]$$.

The charge carrier densities were estimated to be $7.5\times {10}^{18}$ and $1.1\times {10}^{20}$ cm^−3^ for the pristine ZnO NRs and ZnO/Ag_2_WO_4_/AgBr NRs photoelectrodes, respectively. Obviously, ${N}_{d}$ of the ZnO/Ag_2_WO_4_/AgBr NRs photoelectrodes is enhanced, which explains the improved PEC activities of this photoelectrode under solar light compared to pristine ZnO NRs.

### Proposed Mechanism

**Fig. 8:**
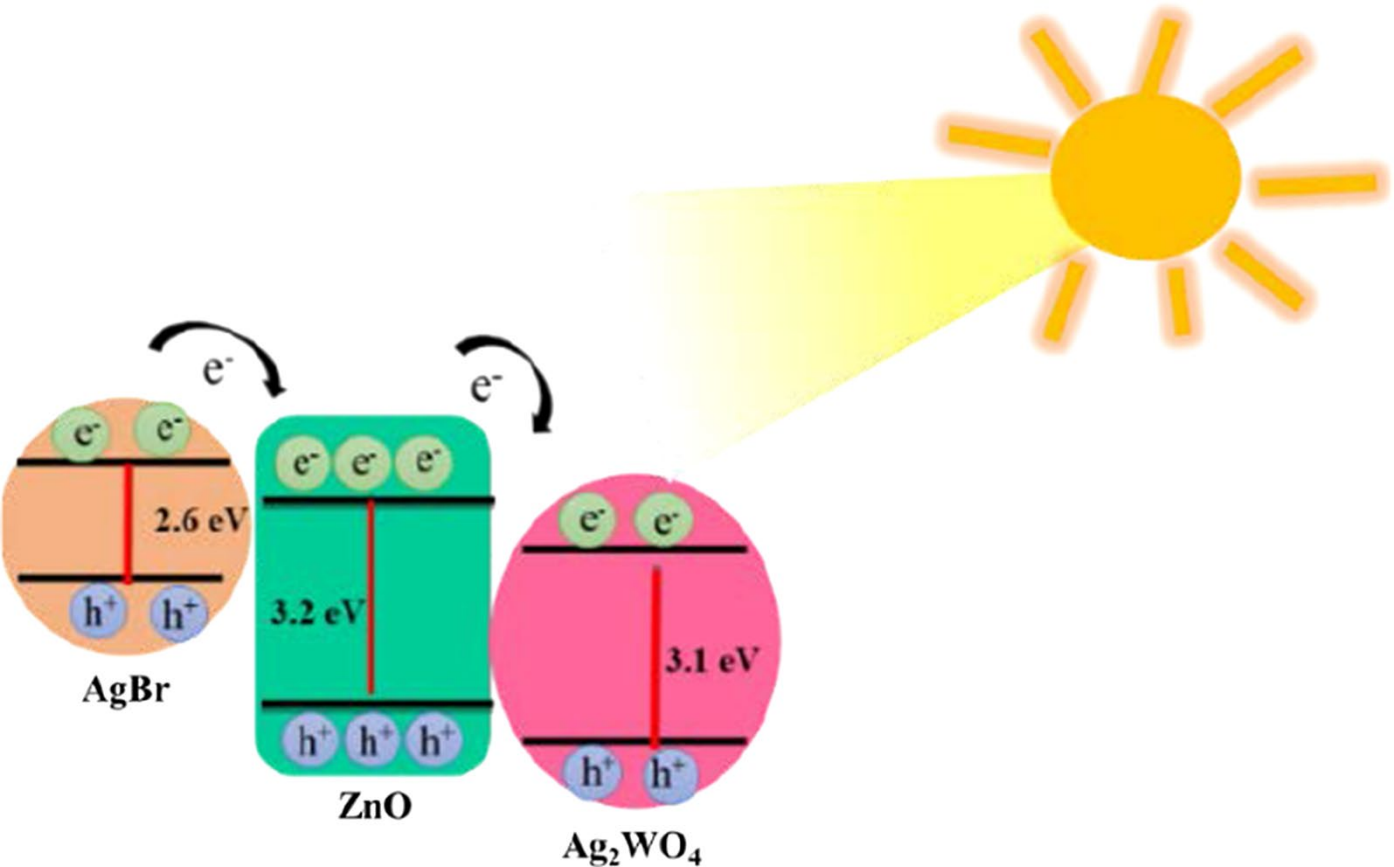
Path transfer of electron–hole in the ZnO/Ag_2_WO_4_/AgBr heterojunction

The energy band position of the ZnO/Ag_2_WO_4_/AgBr heterojunction with possible electron transfer path is illustrated in Fig. 8. Light with lower-energy photons will excite electrons from the VB to the CB of the AgBr due to its suitable band gap (⁓ 2.6 eV), whereas higher-energy photons can excite electrons in the ZnO and Ag_2_WO_4_ semiconductors. Rapid electrons transfers will take place quickly where electrons are transferred from the CB of the AgBr to the CB of the ZnO and Ag_2_WO_4_ consequently. Then, electrons will then be captured and transferred to the photoelectrode contact for reduction reaction in the Pt electrode site where H_2_ should be released. Holes that are left in the VB will perform oxidation reaction and O_2_ should be released. The efficient electrons transfer between the heterojunction can reduce the recombination rate and enhances the photocatalytic reaction.

## Conclusion

In summary, ZnO/Ag_2_WO_4_/AgBr NRs heterostructure photoelectrode was prepared successfully using the hydrothermal growth route followed by the SILAR method. The characterization analysis revealed that the Ag_2_WO_4_/AgBr was successfully deposited onto the ZnO NRs. The photocurrent density of the ZnO/Ag_2_WO_4_/AgBr NRs (10 SILAR cycles) photoelectrode was increased 5 times compared to the ZnO NRs photoelectrode under visible sun radiation. Also, the photoresponse over time showed an improvement in the photocurrent density for the ZnO/Ag_2_WO_4_/AgBr NRs (10 SILAR cycles) photoelectrode (3.3 mA/cm^2^) in comparison with that of the ZnO NRs photoelectrode (1.6 mA/cm^2^). The enhancement in the PEC response is attributed to the synergistic effect due to the deposition of the Ag_2_WO_4_ and the AgBr NPs onto the surface of the ZnO NRs. This deposition increased the absorption of the visible light and the accompanied lowered recombination rate. Also, it was found that higher amount of AgBr can lead to larger aggregates into the heterostructure which might destroy the heterojunction and reduces the PEC performance. The high potential of the ZnO/Ag_2_WO_4_/AgBr NRs photoelectrode for the PEC water splitting makes this photoelectrode a promising candidate for hydrogen production.

## Supplementary Information

**Additional file 1. Figure S1**: Schematic diagram showing the preparation method of the ZnO/Ag2WO4/AgBr photoelectrode, **Figure S2**: XRD patterns of ZnO, ZnO/Ag2WO4 and ZnO/Ag2WO4/AgBr photoelectrodes, **Figure S3**: (a) SEM Image of ZnO/Ag2WO4, (b) SEM Image of ZnO/Ag2WO4/AgBr, (c) EDX spectrum of ZnO/Ag2WO4/AgBr photoelectrode, (d) EDX mapping of the ZnO/Ag2WO4/AgBr heterostructure, and (e) Cross-sectional SEM images and mapping of the ZnO/Ag2WO4/AgBr (10 SILAR cycles) photoelectrode, **Figure S4**: (a) SEM images of the ZnO/Ag2WO4/AgBr with different amount of AgBr loading, **Figure S5**: The high resolution XPS spectrum for Zn, Ag, W and Br collected for the ZnO/Ag2WO4/AgBr sample, **Figure S6**: (a) The plots of (αhν)2 versus hν.(b) Chronoamperometry I-t curves with solar irradiation on/off cycles and (c) LSV curves under dark and visible light conditions of the ZnO NRs, ZnO/Ag2WO4 and ZnO/Ag2WO4/AgBr, **Figure S7**: M-S plots of pristine ZnO NRs, ZnO/Ag2WO4, and ZnO/Ag2WO4/AgBr photoelectrodes, **Figure S8**: Path transfer of electron-hole in the ZnO/Ag2WO4/AgBr heterojunction.

## Data Availability

All data relevant for the reproduction of the results presented in this work are included in this published article or in its supplementary information (SI) file.

## Abbreviations

ZnO: Zinc oxide
Ag_2_WO_4_: Silver tungsten
AgBr: Silver bromide
PEC: Photoelectrochemical
NRs: Nanorods
SILAR: Successive ionic layer adsorption and reaction
XRD: X-ray diffraction
FE-SEM: Field-emission scanning electron microscope
EDX: Energy-dispersive X-ray
XPS: X-ray photoelectron spectroscopy
LSV: Linear sweep voltammetry
M–S: Mott–Schottky
