# Supplementary material for: Solar-Driven Photoelectrochemical Performance of Novel ZnO/Ag2WO4/AgBr Nanorods-Based Photoelectrodes
Source: Nanoscale Res Lett. 2021 Aug 21;16:133. doi: 10.1186/s11671-021-03586-z (PMC8380224; doi:10.1186/s11671-021-03586-z)
Supplement: Supplementary file 1 — Additional file 1. Figure S1: Schematic diagram showing the preparation method of the ZnO/Ag2WO4/AgBr photoelectrode, Figure S2: XRD patterns of ZnO, ZnO/Ag2WO4 and ZnO/Ag2WO4/AgBr photoelectrodes, Figure S3: (a) SEM Image of ZnO/Ag2WO4, (b) SEM Image of ZnO/Ag2WO4/AgBr, (c) EDX spectrum of ZnO/Ag2WO4/AgBr photoelectrode, (d) EDX mapping of the ZnO/Ag2WO4/AgBr heterostructure, and (e) Cross-sectional SEM images and mapping of the ZnO/Ag2WO4/AgBr (10 SILAR cycles) photoelectrode, Figure S4: (a) SEM images of the ZnO/Ag2WO4/AgBr with different amount of AgBr loading, Figure S5: The high resolution XPS spectrum for Zn, Ag, W and Br collected for the ZnO/Ag2WO4/AgBr sample, Figure S6: (a) The plots of (αhν)2 versus hν.(b) Chronoamperometry I-t curves with solar irradiation on/off cycles and (c) LSV curves under dark and visible light conditions of the ZnO NRs, ZnO/Ag2WO4 and ZnO/Ag2WO4/AgBr, Figure S7: M-S plots of pristine ZnO NRs, ZnO/Ag2WO4, and ZnO/Ag2WO4/AgBr photoelectrodes, Figure S8: Path transfer of electron-hole in the ZnO/Ag2WO4/AgBr heterojunction. [file 11671_2021_3586_MOESM1_ESM.docx]

**Supplementary Information**

The figure below is related to Figure 5 in the manuscript:


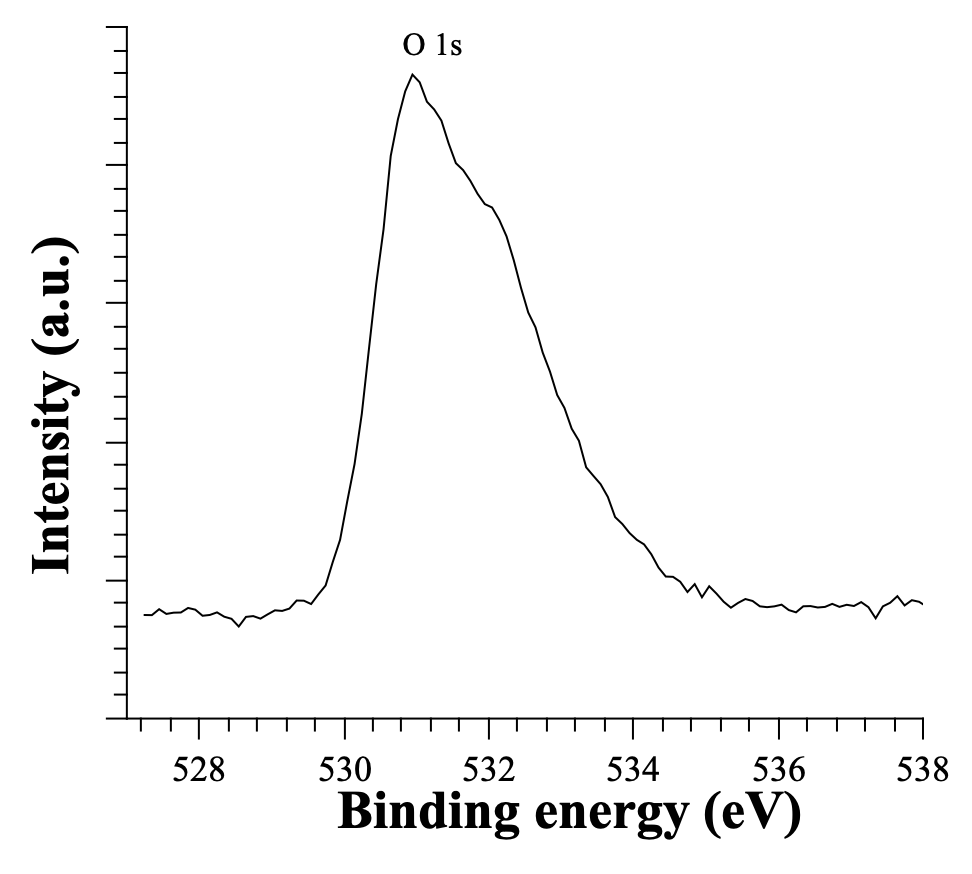


***Figure 1*.** *The high resolution XPS spectrum for O collected for the ZnO/Ag2WO4/AgBr sample.*

The figure below is related to Figure 6 in the manuscript:

***Figure 2.*** *UV-vis absorption spectra*
